# A Smart Multi-Sensor Device to Detect Distress in Swimmers

**DOI:** 10.3390/s22031059

**Published:** 2022-01-29

**Authors:** Salman Jalalifar, Afsaneh Kashizadeh, Ishmam Mahmood, Andrew Belford, Nicolle Drake, Amir Razmjou, Mohsen Asadnia

**Affiliations:** 1 School of Engineering, Macquarie University, Sydney, NSW 2109, Australia; salman.jalalifar@mq.edu.au (S.J.); ishmam.mahmood@students.mq.edu.au (I.M.); andrew.belford@mq.edu.au (A.B.); 2 Faculty of Electrical and Computer Engineering, Shahid Beheshti University, Tehran 1983969411, Iran; Afsaneh.kashizadeh@gmail.com; 3 Smile Like Drake Foundation, Brookvale, NSW 2100, Australia; nikki@solidracks.com; 4 Centre for Technology in Water and Wastewater, University of Technology Sydney, Sydney, NSW 2052, Australia; amir.razmjouchaharmahali@uts.edu.au

**Keywords:** drowning detection, heart rate, oxygen saturation, accelerometer, water depth, time of submersion, adjustable threshold values

## Abstract

Drowning is considered amongst the top 10 causes of unintentional death, according to the World Health Organization (WHO). Therefore, anti-drowning systems that can save lives by preventing and detecting drowning are much needed. This paper proposes a robust and waterproof sensor-based device to detect distress in swimmers at varying depths and different types of water environments. The proposed device comprises four main components, including heart rate, blood oxygen level, movement, and depth sensors. Although these sensors were designed to work together to boost the system’s capability as an anti-drowning device, each could operate independently. The sensors were able to determine the heart rate to an accuracy of 1 beat per minute (BPM), 1% SpO_2_, the acceleration with adjustable sensitivities of ±2 g, ±4 g, ±8 g, and ±16 g, and the depth up to 12.8 m. The data obtained from the sensors were sent to a microcontroller that compared the input data to adjustable threshold values to detect dangerous situations. Being in hazardous situations for more than a specific time activated the alarming system. Based on the comparison made in the program and measuring the time of submersion, a message indicating drowning or safe was sent to a lifeguard to continuously monitor the swimmer’ condition via Wi-Fi to an IP address reachable by a mobile phone or laptop. It is also possible to continuously monitor the sensor outputs on the device’s display or the connected mobile phone or laptop. The threshold values could be adjusted based on biometric parameters such as swimming conditions (swimming pool, beach, depth, etc.) and swimmers health and conditions. The functionality of the proposed device was thoroughly tested over a wide range of parameters and under different conditions, both in air and underwater. It was demonstrated that the device could detect a range of potentially hazardous aquatic situations. This work will pave the way for developing an effective drowning sensing system that could save tens of thousands of lives across the globe every year.

## 1. Introduction

It is estimated that every year, 372,000 people around the world die due to drowning, and drowning is one of the top 10 unintentional causes of death [1]. As stated by the “National Drowning Report” published by Royal Life Saving Australia, there was a significant 20% increase in the number of drownings from 2020 to 2021 [2]. Many factors could lie behind drownings, including pre-existing medical conditions such as cardiac complications and drug or substance abuse [3,4,5,6]. However, the most common drowning causes are an inability to swim and panic in the water. In addition, unattended and unsupervised children comprise a substantial portion of the victims [7].

Drowning tends to be silent, and victims rarely move in a convulsive manner. Instead, they expend significant energy trying to keep their heads above water and may be unable to call or signal for help. If water meets their larynx or tracheae, they will panic and go into spasm, which prevents them from shouting for help. Normally, the only way to detect drowning is finding the victim struggling in the water with difficulty breathing or an irregular heartbeat. Victims can remain upright and, unless rescued by a trained lifeguard, may struggle on the surface of the water for only 20 s to 60 s before immersion occurs. Therefore, when a person is missing in the water, the time taken to locate them is critical to their survival.

There are a wide variety of solutions to prevent people from drowning, such as close monitoring, training and/or educating, as well as teaching basic swimming rules, regulations, and etiquette. Although these solutions have proven to be quite useful in many ways, they are not as effective they might be. Thus, developing an accurate device capable of detecting a dangerous situation during which a swimmer is having trouble in the water or is on the verge of drowning would ensure a dramatic decrease in water-related deaths and accidents. In addition, such devices would save lives and reduce the amount of money spent on search and rescue.

### Related Works

Based on previous research, drowning detection systems can be categorized into two major classifications: sensor-based and image processing systems. The former uses sensors such as pressure, heartbeat, motion, and depth, and the latter applies multiple algorithms to detect drowning through capturing images from live videos. Shehata et al. [8] compared different drowning detection methods by assessing the accuracy levels, complexity, and costs involved for the mentioned systems. In terms of cost and complexity, sensor-based devices are categorized in low to moderate classifications, whereas image processing systems are considered complex and expensive [8]. They often require drones to cover a wide area involving safety complications and practical challenges such as charging batteries for the drones. The accuracy is the only aspect where image processing techniques outperform sensor-based systems [8]. However, this is not the case when a drowning occurs underwater where the swimmers are not visible in murky water. There have been increasing attempts on developing sensory systems in the form of wearable devices to detect drowning. Chaudhari et al. [9] developed a device in which the heart rate is monitored and compared to a given threshold value and then transmitted using a radio with a range of 5–6 m [9]. The entire system can be attached to the swimmer’s head or hand, allowing easy mobility [9]. John et al. [10] proposed another module-based systems consisting of a heart rate pressure sensor. Recently, there has been a significant progress on developing sensitive sensors with small fingerprints which has potential for using in drowning detection systems [11,12,13,14,15,16,17]. For the transmitter side, the wristband design consists of a microcontroller together with a heart rate sensor, with two pre-defined thresholds for heart rate readings, i.e., high and low. Similarly, to the previous two-module designs, if a heart rate reading above the threshold values is recorded, an alert is sent to the receiver. A three-module system to detect drowning is presented by Ramdhan et al. [18]. The system is split into monitoring, access point, and drowning detection segments. The drowning detection module comprises a microcontroller with a pulse sensor to measure the heartbeat. The method proposed in [19] utilizes three sensors: an oxygen saturation level sensor, a respiration monitoring sensor, and a water sensor. These sensors are used for measuring parameters such as blood oxygen saturation levels, respiratory movements, and the submersion of a person’s body underwater, respectively, processed by a controller.

Simple sensor systems paired with airbags have also been widely researched. For example, Geetha et al. [20] demonstrated an automatic drowning prevention system with a PIC (peripheral interface controller) integrated with an accelerometer. When the threshold values are surpassed, the wristband device will automatically open an airbag, which will permit the victim to float. Table 1 provides a list of recent studies and compares their performances.

**Figure 1:**
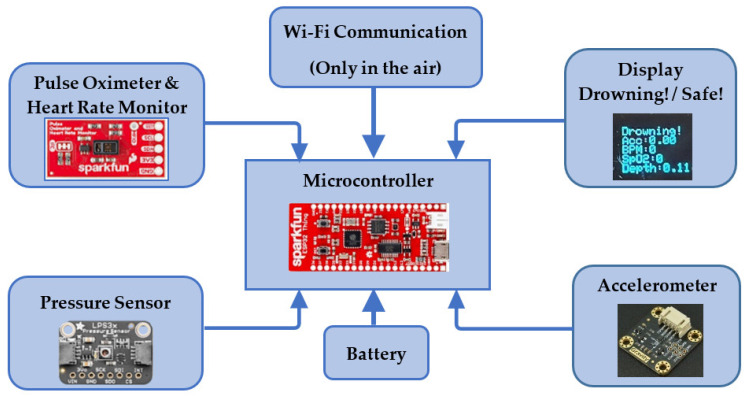
Components of the drowning detection system.

This paper suggests a fully functional, waterproof drowning detection wearable device. The device measures four parameters: heart rate, blood oxygen saturation, acceleration and water depth. The data obtained from each sensor is processed by the microcontroller, in which the sensors’ data is compared to pre-defined (adjustable) threshold values. If any of the measured values cross the corresponding threshold for longer than a pre-defined time, a “Drowning” message is shown on display regardless of the status of other sensors. Besides, the board is also equipped with Wi-Fi that can send signals to a specific IP address reachable via mobile phone or laptop. The overall block diagram of the proposed system is illustrated in Figure 1.

## 2. System Design

Overall, an average healthy adult can hold his or her breath for approximately 30 s, but for children, the duration is less [21]. Therefore, there is only a short time to take action to save a potential victim of drowning. This clinical reality reveals the importance of the quick detection of the determining parameters of distress. In order to detect a drowning situation, finding the decisive factors is vital. Since the environmental parameters such as water quality and air and water interfaces are very different, it is suggested to work on certain body factors.

### 2.1. Measurable Parameters

Although there are several commonplace signs for drowning, only a few are measurable parameters and always appear in every scenario [22]. Based on a study conducted in New England, heart rate and oxygen level are two biometric factors by which a victim of near-drowning needs to be assessed [23]. Depth, movement pattern and duration of submersion are other important factors examined in this paper to prevent drowning.

#### 2.1.1. Heart Rate

In a very early study conducted by the University of Toronto, researchers found that the heart rate (HR) patterns in different categories of drowning victims are the same [24]. It was noted that in both cases, the heart rate dropped significantly and noticeably in the moments leading up to death [24]. In this regard, Norwegian researchers carried out a case study in which it was revealed that within minutes of submersion, the individual’s heart rate dropped to 43 Beats per minute (BPM) and fluctuated within a steady rate between 35 BPM and 78 BPM [25]. By analyzing the data obtained from this case, the assumption was drawn that for testing purposes of our system, a threshold heart rate of equal to or less than 55 BPM would be cause for concern. This implies that drowning is detected when the heart rate value is 55 BPM or less, and the corresponding message is displayed on the system.

#### 2.1.2. Blood Oxygen Saturation

The significant decrease in heart rate leads to a subsequent drop in blood oxygen saturation. This happens due to the decrease in the heart’s ability to pump oxygenated blood into the body. So, blood oxygen saturation (SpO_2_) is the next determining factor. SpO_2_ is of extreme importance, as if its level drops below a certain value, the body will be unable to carry out its normal functions. The normal range of oxygen saturation in the human body is a consistent value of 95–100% [26], and any values below this number will be considered as a sign of hypoxia. Predictably, when a person is undergoing the process of drowning, his or her oxygen level will fall dramatically, so the oxygen level in the individual’s bloodstream is a suitable indicator of whether individuals are experiencing suffocation from drowning.

#### 2.1.3. Movement Pattern Recognition

A LIS2DH accelerometer (Core Electronics, Adamstown, NSW, Australia) is used to track the swimmer’s movement pattern. It allows monitoring the swimming behavior, including the situations when the swimmer does not move for a certain time or moves quickly as a sign of distress. For example, the sensor can detect if the individual is still moving or immobile. If the swimmer does not move for a defined period of time (i.e., 10 s), the microcontroller can be defined to send a “drowning” signal to the OLED (Organic Light-Emitting Diode) display. Besides, at the onset of the drowning, the swimmer’s movement pattern may change drastically due to irregular paddling. Although the abrupt changes in swimming patterns can be an excellent indicator to reveal the drowning, no specific values of this parameter have been reported as an indicative threshold value, and further study is still required to obtain its values. Movement pattern sensors, together with other sensors, can be used to make decisions as to whether the swimmer is in a distress situation or not.

#### 2.1.4. Water Depth

The rescue history of 56,000 cases in a lifeguard-staffed waterpark environment revealed that depth is a very critical parameter indicating drowning [27]. It was seen that 42% of the total rescues occurred in waters of depth equal to or less than 1.52 m (shallow water), and 56.6% of rescues happened in depths greater than 1.52 m (deep water) [27]. Thus, it is imperative to have an option of measuring depth in the proposed device. In this study, the water depth threshold can be set to any desired values, which means that if the depth in which the person is swimming is greater than the defined value for more than a given period, drowning will be indicated. As with the other threshold values, it is possible to change the depth threshold based on the swimmer’s surroundings.

#### 2.1.5. Duration of Submersion

As an individual can be drowned within 60 s with as little as half a cup of water entering the lungs [28], a time duration of approximately 15 s is considered as a pre-defined threshold value in our study. Therefore, if any of the indicators mentioned above (heart rate, SpO_2_, Acc, and water depth) are triggered continuously for more than 15 s, it would indicate drowning, and the respective rescue message will be signaled. Once the necessary factors for drowning detection are determined in this section, the most appropriate sensors for measuring the values of the mentioned parameters are investigated in the next section.

### 2.2. Sensing Elements

#### 2.2.1. Pulse Oximetry

As mentioned before, heart rate and blood oxygen saturation are two important biometric parameters measured in our system. As both the parameters are closely related in terms of measurability, a MAX30101 (SparkFun Electronics, Boulder, CO, USA) sensor was chosen to measure both simultaneously. A Pulse oximeter measures blood oxygenation and heart rate noninvasively and accurately [29]. In order to employ the oximeter sensor, a compatible breakout board is required. SparkFun was assessed to be the most viable among the various existing options. It is a well-reputed manufacturer whose breakout board comprises the MAX30101, and implementation of the sensors is allowed via Qwiic cables. Qwiic cables provide sturdy and robust connections, which is difficult to achieve with soldering or fly wires.

**Figure 2:**
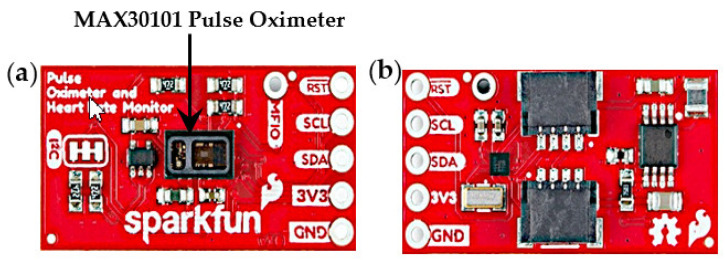
(**a**) SparkFun MAX30101 pulse oximeter and heart rate monitor; (**b**) back view showing Qwiic connectors (Adapted from [23]).

As seen in Figure 2, the MAX30101 is placed at the center of the breakout board, where the finger/wrist was placed to obtain readings. The SparkFun biometric sensor communicates with the microcontroller using I2C communication [30]. The SparkFun pulse oximeter and heart rate monitor has an operating voltage of 3.3 V [30]. Its dimensions are 25.4 mm × 12.7 mm, which is compatible with the project requirement of a compact sensor [30].

#### 2.2.2. Pressure Sensor

The pressure exerted by the surrounding environment of a swimmer directly impacts his drowning. Thus, a sensitive sensor is required to measure the pressure. There are six primary technologies for implementing pressure sensors: potentiometric, inductive, capacitive, piezoelectric, strain gauge and variable reluctance pressure sensors [31]. However, as per research conducted by A. Mohan et al. [31], piezoresistive Microelectromechanical Systems (MEMS) are the most feasible sensors for measuring pressure in deep and harsh underwater environments. They feature a flexible membrane that acts as the spring element for pressure sensing. As a result, these pressure sensors are capable of not only withstanding an extremely high value of pressure but also simultaneously obtaining accurate readings at varying temperatures. The pressure sensor selected for this study is LPS33HW (Adafruit, New York, NY, USA). It can resist very high pressures up to 1260 hPa (12.8 m of water) and a wide range of temperatures from −40 °C to +85 °C. It is also waterproof, courtesy of a protective gel lining inside the integrated circuit, and resistant to the abrasive chemicals generally found in swimming pools.

The LPS33HW can communicate via both I2C and SPI (Serial Peripheral Interface) methods of communication. Since soldering on such a small-scale pressure sensor is very difficult, the Adafruit LPS33HW breakout board was chosen for this project. The Adafruit LPS33HW consists of the LPS33HW sensor mounted on a breakout board and is small and compact. It has the option of connection via Qwiic cables and can register and display pressure readings with an accuracy of ±0.1% hPa. The highest possible pressure reading is equivalent to a 12.8 m depth of water, but the sensor is able to withstand pressure values up to 20 times the given measurement range. This feature is also useful to our proposed system, as it allows the system to apply to extreme depths of water bodies. The operating voltage of this sensor is compatible with that of the oximeter at 3.3 V. The sensor dimensions are 25.7 mm × 17.7 mm × 4.6 mm, and it is connected to a microcontroller using I2C communication.

**Figure 3:**
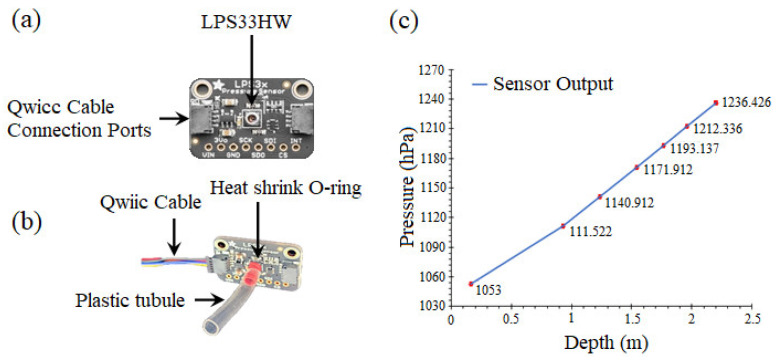
(**a**) Adafruit LPS33HW pressure sensor; (**b**) Tubule and Qwiic cable connections; (**c**) LPS33HW pressure sensor performance. Red marks are the values of standard deviation (STDEVs).

The Adafruit LPS33HW is shown in Figure 3a. The tubule on the LPS33HW allows the attachment of an external tube through it, and the O-ring allows the sensing element to protrude from the device surface, a feature that is of great importance in the overall design of the proposed system. In order to test the functionality of the sensor, the existing tubule was extended by a heat-shrink tube (Figure 3b), and steady pressures are exerted upon it by a calibrated pressure gauge. The pressure sensor performance was examined by pressurizing it using a vertical tube filled with varying amounts of water. Illustrated in Figure 3c, the red marks are standard deviation values (STDEVs). As STDEVs are in the order of one-hundredth of a percent, the error bars are not distinguishable on the measured pressures. In summary, the sensor can produce an output value that closely matches the expected value and is sufficiently accurate for our application.

#### 2.2.3. Microcontroller

The microcontroller implemented in this paper was ESP32 Thing (SparkFun Electronics, Boulder, CO, USA). This board operates on a 3.0–3.6 V voltage range and can be powered by a micro-USB port and a lithium battery. It also has an integrated Wi-Fi transceiver suitable for IoT (Internet of Things). The board dimensions are 59 mm × 25 mm, suitable for prototyping. It can also be replaced by smaller compatible devices when required.

#### 2.2.4. Battery

In order to conduct underwater tests, portability is an essential requirement, enabled in this work by using a battery for the power supply. The discharge rate of the battery (C rating) is a determining factor for battery selection. A C rating of 2 was sufficient for our device, as all the tests could be conducted within 30 min. The critical criterion here for the battery is its weight which should be minimized.

Thus, a lithium–polymer (LiPo) battery (Core Electronics, Adamstown, NSW, Australia) was chosen for the system. This battery is extremely lightweight, has reasonable discharge rates and is not sensitive to the changes in pressure to which this device would be subjected. The battery supplies 3.7 V and has a current rating of 1100 mAh, with a discharge rating of 2C. In addition, it has a built-in protection circuit to ensure over-drawing, and voltage fluctuations do not occur. The overall weight of the battery is 21 g, and it can withstand temperatures between −10 °C and 50 °C.

#### 2.2.5. OLED Display

Since the current prototype has only an integrated Wi-Fi on the board, the only wireless communication is in the air, and it cannot communicate wirelessly underwater. Thus, a screen is needed to display the data obtained from the sensors. The requirements for the screen revolve around its dimensions and connections. Therefore, a Qwiic micro-OLED screen (SparkFun Electronics, Boulder, CO, USA) was used.

#### 2.2.6. Connections and Communication Protocols

**Figure 4:**
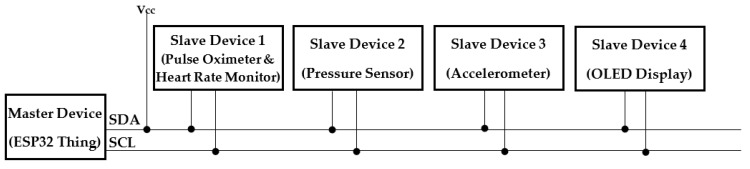
I2C Communication protocol in the proposed system.

Most of the connections within the device were carried out using Qwiic cables. It eliminated the requirement of soldering; thus, the systems could be more robust and flexible at the same time. The maximum current drawn through a Qwiic cable was 226 mA, which was sufficient for our system. The communication protocol implemented in our system was I2C communication. The ESP32 Thing served as the master, and the pulse oximeter, the heart rate monitor, the pressure sensor, the accelerometer, and the OLED display were the slave devices (Figure 4). The maximum distance over which I2C communication could be made was 1 m, which fell within the constraints of our system. As pull-up resistors were already embedded into the breakout boards, there was no need for further resisters to be included in the communication wiring.

#### 2.2.7. Power Usage and Range

The device employed a pulse oximeter and heart rate sensor, a pressure sensor, an accelerometer, and an OLED display. The pulse width of the OLED display can be changed in the program over a the range of 69–411 µs which allows the algorithm to optimize SpO_2_ and HR accuracies and power consumption based on various usage. Furthermore, the operating voltage of the sensor was 3.3 V. As a result, the power usage of the heart rate monitor was as low as <1 mW. The Adafruit LPS35HW pressure sensor could be used for either the operating voltage of 3 V or 5 V. The operating voltage of the accelerometer was 3.3–5 V, and its operating current was 8–10 µA in the low-power and low-noise mode) and 0.12 mA in the high-performance mode. The operating voltage range of the ESP32 Thing was 2.2–3.6 V, and its operating current was 150 mA when the Wi-Fi signal was active. The operating voltage of the OLED display was 3.3 V, and the maximum operating current was 100 µA. A 1100 mAh LiPo battery supplied the power of the waterproof portable device.

The transmission distance of the Wi-Fi signal in the air was the same as that of any typical personal Wi-Fi network. However, wireless communication using LoRa, which is currently in the testing procedure stage, was higher underwater and 20 m above the water. This range can be scaled up in the future.

## 3. Hardware Integration of the System

**Figure 5:**
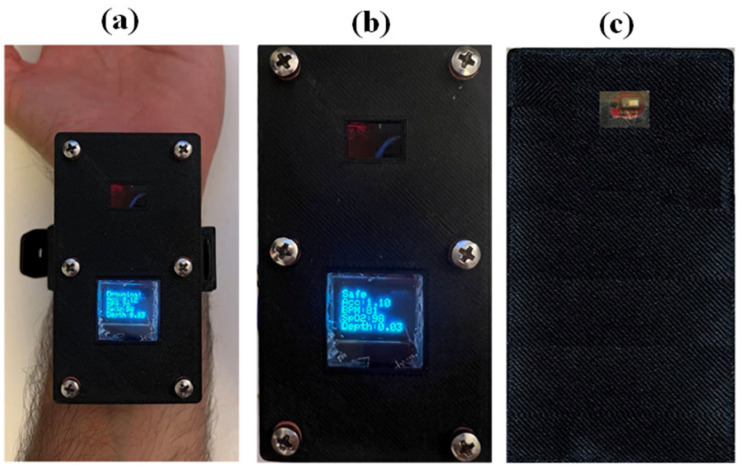
(**a**) device worn around the wrist; (**b**) top view of the device; (**c**) bottom view of the device.

The practical implementation of the system can be seen in Figure 5 (Supplementary Materials). The dimensions of the device are 80 mm × 40 mm × 35 mm. The pulse oximeter and heart rate monitor were mounted on the bottom surface of a box to touch the swimmer’s wrist to detect the swimmer’s heart rate and blood oxygen saturation. The pressure sensor was attached to a sidewall where the tubule touched water through a hole and measured the water depth. The OLED screen was fixed to the lid of the box to allow the user to read the screen easily. The accelerometer was also attached to the sidewall of the box. The battery was placed at the bottom of the box, and then, the board was placed on top of it. Finally, all parts were connected to the board through suitable wiring.

## 4. Working Principle

**Figure 6:**
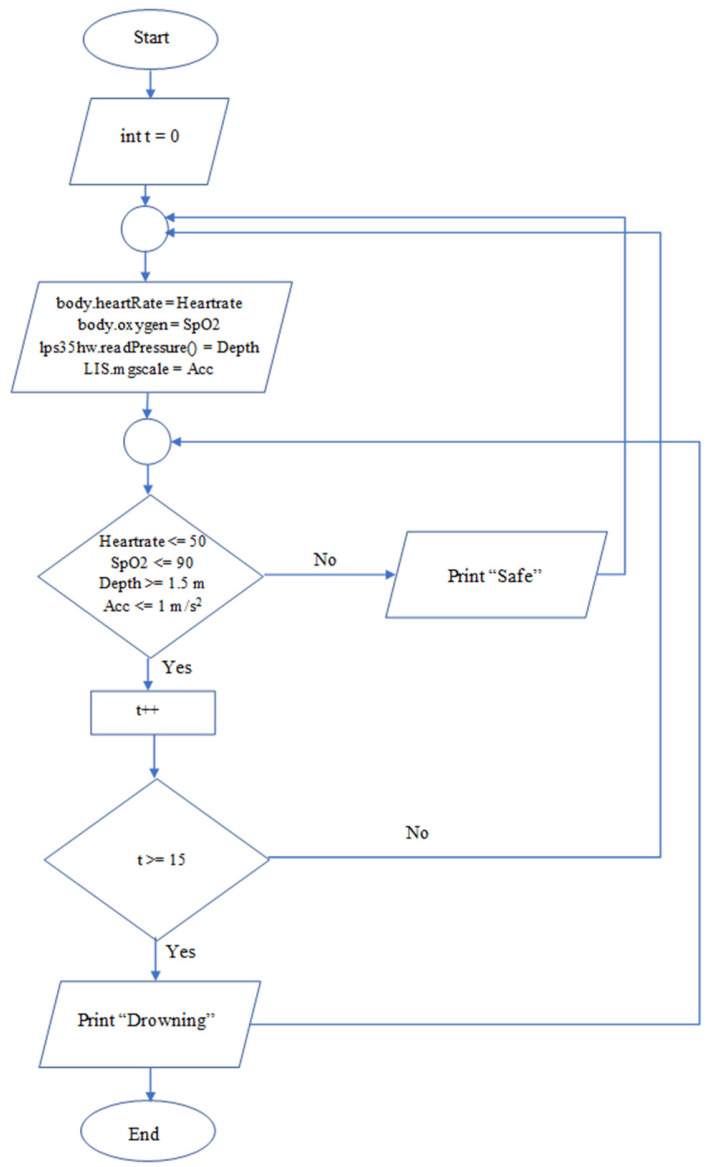
Flowchart of the code implemented into the proposed system.

The heart rate, the blood oxygen saturation, the acceleration, and the water pressure are the parameters measured by sensors for our system. These data are sent to the microcontroller for processing. A specific threshold value for each sensor can be adjusted based on the users’ overall health conditions. The received data are compared continuously to these predefined setpoints. If any threshold values are reached, a timer is triggered in the system. The function of this timer is to measure the amount of time for which the received value is higher than the threshold. If the threshold values are exceeded for longer than the pre-set time, a dangerous condition will be recognized, and a drowning message will appear on display. Once the received data are returned to the safe range, the timer will be reset and not initiated until another sensor records a threshold value. Their threshold values are set so that they are accurate indications of dangerous situations. The system-coding flowchart is illustrated in Figure 6.

## 5. Methodology, Results, and Discussion

To ensure the system functionality, several tests were conducted, which are fully discussed in the following section.

### 5.1. Testing Sensors Individually

After integrating the whole system, each sensor was subjected to various conditions to confirm it operated correctly. Each sensor was tested, while the values of the other sensors were kept constant.

#### 5.1.1. Heart Rate Test

**Figure 7:**
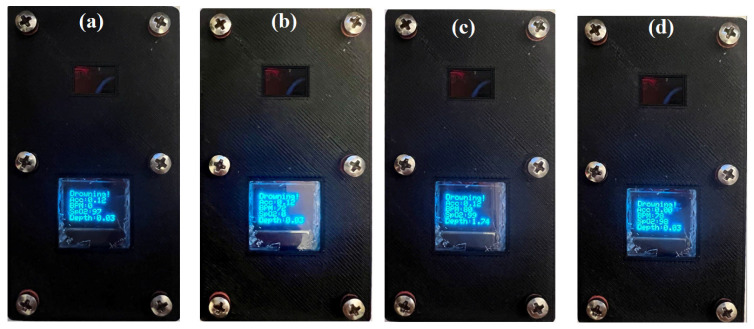
Drowning is indicated due to (**a**) the low heartrate, (**b**) the low SpO_2_ level, (**c**) the high pressure/depth, (**d**) and low acceleration.

In the prototype system, the threshold value for the heart rate was set at 50 BPM. The value of 50 BPM is a generalized, all-rounded value based on the data obtained from near-drowning victims [24]. This means that if the device detects a heart rate of less than or equal to 50 BPM for more than a certain amount of continuous time, the display will show a “Drowning!” message. To test the sensor, one finger (or wrist) was pushed on the sensing element, so that the sensor could not read the heart rate and a drowning message was shown, as seen in Figure 7a.

#### 5.1.2. Blood Oxygen Saturation

The threshold value was set at 90% to test the device functionality for detecting low blood oxygen saturation. Any recorded value less than or equal to 90% for a given amount of continuous time will indicate that the swimmer is on the verge of drowning. For example, a drowning indication due to a low SpO_2_ level can be seen in Figure 7b. In this case, the finger was placed on the sensing element more gently to read the heartbeats, but SpO_2_ could not be detected.

#### 5.1.3. Water Depth

The pressure sensor was responsible for measuring the depth of submersion. As stated before, drowning can occur in depths ranging from shallow waters to deeper water bodies and does not necessarily have to be in bottomless waters. Based on the research [27], the threshold value for water depth was 1.5 m. It means that if a depth of greater than 1.5 m was sustained for more than a given time, then drowning would be indicated. For example, Figure 7c shows a depth of 1.74 m, which was achieved by manually applying a high-test pressure. The result illustrated the pressure sensor independent nature and the sensor ability to withstand and record high pressures.

#### 5.1.4. Swimmer Movement

The accelerometer (LIS2DH) was responsible for monitoring the swimmer movement pattern. As mentioned earlier, the swimmer’s movement pattern may change drastically due to varying paddling at the beginning of the drowning. Although the sharp changes in swimming patterns can be an excellent indicator to indicate drowning, no specific values of this parameter have been reported as an indicative threshold value, and further research is needed to acquire its significances. On the other hand, in many cases, the swimmer will have a uniform and reduced movement after a drowning accident. The accelerometer measures the gravitational acceleration. If this is subtracted from the total detected by the sensor, the zero or base-line value can be a good reference against which determine drowning. Therefore, in this paper, a small value of 1 m/s^2^ was considered the threshold value of the accelerometer to show drowning. For instance, Figure 7d shows an Acc of zero, achieved when the device is stationary. The result illustrated the impact of the accelerometer individually to detect the swimmer’s movement pattern.

### 5.2. Testing the Entire System

Once all the components were tested out separately, the testing process was conducted for the entire system in both air and water. The tests aimed to evaluate the entire system functionality in different conditions and over various time periods.

#### 5.2.1. Air Test

**Figure 8:**
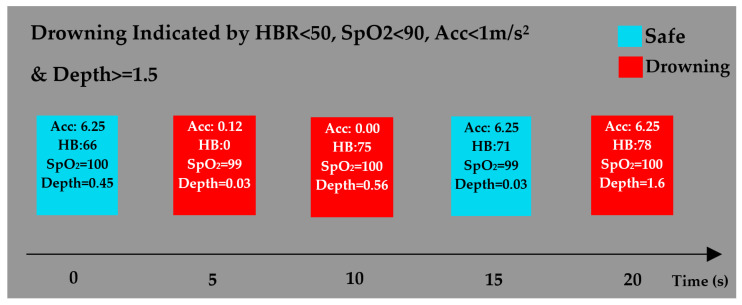
Drowning is indicated by the heart rate lower than 50 beats per minute (BPM), the SpO_2_ level less than 90%, the acceleration lower than 1 m/s^2^, and the depth greater than 1.5 m for more than 5 s.

For these tests, the pressure was applied manually using a syringe. Various heart rates and blood oxygen saturation values were obtained for the subject by doing light to rigorous exercise. Figure 8 illustrates the initiation of a drowning message over a time span of 15 s. The system was set to detect drowning for the heart rate of <50 BPM, the SpO_2_ level of <90%, the water depth of ≥1.5 m, or the acceleration of <1 m/s^2^ for more than 15 s.

As demonstrated in Figure 8, drowning is indicated at the second- and third-time frames as the heart rate and the Acc were recorded at less than 50 BPM and 1 m/s^2^, respectively, for the entire 5 s interval. Following that, the Acc returned to 6.25 m/s^2^, so the fourth reading did not denote drowning. The pressure, however, increased over the 5 s interval. It increased the depth to a value over 1.5 m; thus, the last reading indicated drowning.

**Figure 9:**
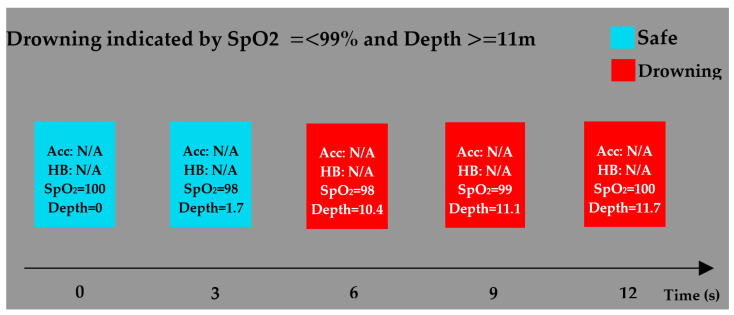
Drowning is indicated by the SpO_2_ level of ≤99% and the depth of ≥11 m for 3 s.

In another experiment, the drowning condition was investigated for readings of the SpO_2_ level of ≤99% or a pressure reading of ≥11 m (Figure 9). In the second frame, a change in SpO_2_ was noticed, whereas the pressure increased slightly. Drowning was indicated during the third set as the blood oxygen saturation was less than 99% for more than 3 s, even though the depth was still below the defined threshold value. Both thresholds were met in the fourth set of readings; thus, drowning was still indicated. In the final set of readings, the blood oxygen saturation increased to 100%, but drowning was still indicated as the depth was at a value of 11.7 m, which was 0.7 m above the threshold. This experiment demonstrated that the system has the ability to work independently based on its readings, but it is also capable of recording extreme depths and is most definitely applicable for underwater applications.

#### 5.2.2. Underwater Test

**Figure 10:**
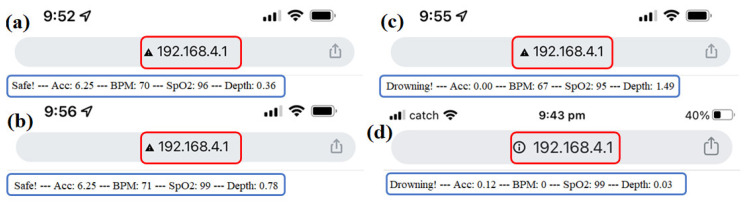
The indicated signals transferred via Wi-Fi to a mobile phone which was read by accessing the IP address “192.168.4.1”, (**a**) the “safe” message is shown since Acc > 1 m/s^2^, BPM > 50, SpO_2_ > 90, and Depth < 1 m, (**a**,**b**) shows the “Safe” message, since Acc > 1 m/s^2^, BPM > 50, SpO_2_ > 90, and Depth < 1 m, (**c**) shows the “Drowning” message, since Acc < 1 m/s^2^ and Depth > 1 m, and (**d**) shows the “Drowning” message, since Acc < 1 m/s^2^ and BPM < 50.

In this section, the operation of the device in a swimming pool is discussed. Underwater tests were conducted in a conventional public swimming pool. Since the pool is about 1.6 m deep, the threshold value for the depth was temporarily set to 1 m so that any depth equal to or beyond 1 m would indicate drowning. As shown in Figure 10, the sensor outputs, and the swimming condition (safe or drowning) could be sent to a smartphone or laptop wirelessly. As seen, Figure 10a,b shows the “Safe” message, since Acc > 1 m/s^2^, BPM > 50, SpO_2_ > 90, and Depth < 1 m. Figure 10c shows the “Drowning” message, since Acc < 1 m/s^2^ and Depth > 1 m, and Figure 10d shows the “Drowning” message, since Acc < 1 m/s^2^ and BPM < 50.

**Figure 11:**
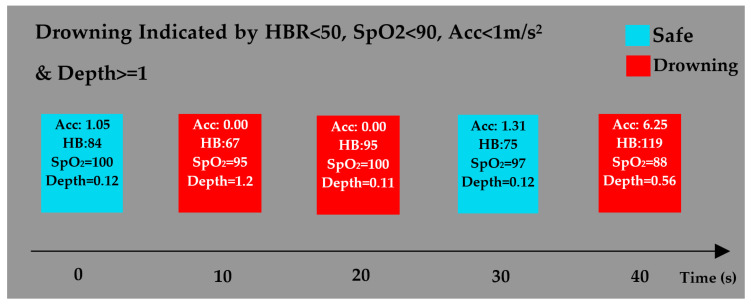
Drowning is indicated by the heart rate lower than 50 BPM, the SpO_2_ less than 90%, the acceleration lower than 1 m/s^2^, and the depth greater than 1 m for more than 10 s.

Figure 11 also shows the swimming pool underwater test conditions revealed on the OLED display. In this case, the drowning condition was examined for readings with the SpO_2_ level of ≤90%, the heartbeat rate of <50 BPM, the pressure reading of ≥1 m, or the acceleration of <1 m/s^2^. As shown, when the device was outside water, it sent a “safe” signal to the IP address since the indicated values for acceleration, heartbeat, SpO_2_, and depth was 1.05 m/s^2^, 84 BPM, 100%, and 0.12 m, respectively and all these values are in the safe mode. As mentioned earlier, these signals were also displayed on the OLED display. However, no signals were sent to the IP address underwater, and the indicators values were readable through the OLED display. In the second scenario, the swimmer held the device underwater, below the 1 m depth, while stationary. Thus, the drowning signal was displayed on the OLED display. Although the device was near the water surface in the third test condition, it was still not moving. That is why the drowning signal was displayed again. The swimmer moved his arms outside the water with a smooth Acc in the fourth test condition. Thus, the “safe” signal was displayed. Finally, in the fifth test condition, the swimmer moved underwater, holding his breath for a while until his heartbeat rate increased, whereas his SpO_2_ decreased below the threshold value. In this case, the “drowning” signal was displayed on the OLED display.

## 6. Conclusions

In the proposed anti-drowning system, it was observed that the sensors measuring the heart rate, the oxygen level saturation, the acceleration, and the water depth could collaborate efficiently while performing their functions independently. Based on the tests conducted in air and water, the proposed system has a great capacity to extract and read data accurately, even in harsh environmental conditions.

The underwater tests demonstrated that the system has the potential to read values whilst submerged, as well as in the air. Furthermore, according to the underwater experiments, the system can detect and display changes in a water depth of 0.01 m.

Overall, the proposed system can be considered a fully functioning prototype capable of detecting and preventing drowning in swimmers. However, this system has much potential for practical and commercial implementation. Initially, a communication system will be created for a short distance to the surface, and then, attached or free-floating transponders will be provided to forward the message to the mobile communication network, allowing the system to send out distress signals to the rescuer. The second enhancement is to reduce the size of the components and packaging, so that it is practical as a wearable device. Finally, the development of a mobile application for the system, which would allow the user to enter specific details about any pre-existing heart conditions or health issues or information regarding the depth of the water body into which the swimmer is about to go, could also be included.

## Figures and Tables

**Table 1:** Recent studies that are conducted on sensor-based drowning detection systems.

Reference	Sensing Elements	Transmitter Circuits	Receiver Circuits	Working Principle	Attachment Area
Chaudhari et al. [9]	Heartbeat rate sensor	ATmega 328 microcontroller RF ^i^ module Regulator IC ^ii^ LCD ^iii^ display Rechargeable battery Push button switches to determine limitations for pulse counts	An ATmega 328 microcontroller RF module Transformer Regulator Rectifier Buzzer	Comparison of the obtained value with the given thresholds	Hand or head
John et al. [10]	Heart rate pressure sensors	Arduino Lilypad microcontroller 433 MHZ transmitter module	A 433 MHZ receiver Arduino Uno microcontroller Buzzer LCD display	Comparison of the obtained value with the given thresholds measurement of the submerging time.	Wrist
Ramdhan et al. [18]	PPG ^iv^ types of pulse sensor	An Arduino Pro Mini 328 433 MHz (UART) transmitter module	A 433 MHz (UART) transceiver module Raspberry Pi 2, A cloud network	Monitoring of the real positions of swimmers via a mobile app or web calculation of the time signal lost under the water comparison of the calculated time to a predefined value.	Head
Kulkarni et al. [19]	Water sensor Respiratory monitoring system (RMS) Muscle oxygen saturation sensor (MOSS)	ATMEGA 32A controller		Comparison of the obtained values of each sensor with the given thresholds Indication of drowning if at least 2 of the sensors detect drowning independently	Arm
Geetha et.al. [20]	Accelerometers	A PIC16F877A microcontroller GSM ^v^ module Battery Inflator Airbag	GSM module	Opening of a small airbag system when the device senses the movement of the person who is in danger	Wrist

^i^ Radio frequency; ^ii^ Integrated Circuit; ^iii^ Liquid Cristal Display; ^iv^ Photoplethysmography; ^v^ Global System for Mobile Communications.

## Data Availability

The data used to support the findings of this study are available from the corresponding author upon request.

## Supplementary Materials

The following supporting information can be downloaded at: https://www.mdpi.com/article/10.3390/s22031059/s1.
